# ReIMAGINE: a prostate cancer research consortium with added value through its patient and public involvement and engagement

**DOI:** 10.1186/s40900-021-00322-w

**Published:** 2021-11-17

**Authors:** S. Green, S. Tuck, J. Long, T. Green, A. Green, P. Ellis, A. Haire, C. Moss, F. Cahill, N. McCartan, L. Brown, A. Santaolalla, T. Marsden, M. Rodriquez Justo, J. Hadley, S. Punwani, G. Attard, H. Ahmed, C. M. Moore, M. Emberton, M. Van Hemelrijck

**Affiliations:** 1 Translational Oncology and Urology Research (TOUR), School of Cancer and Pharmaceutical Sciences, King’s College London, London, UK; 2 ReIMAGINE Consortium Patient Representative, London, UK; 3 UCL Division of Surgical and Interventional Sciences, University College London, London, UK; 4 Department of Urology, University College London Hospitals NHS Foundation Trust, London, UK; 5 MRC Clinical Trials Unit, University College London, London, UK; 6 Centre for Medical Imaging, University College London, London, UK; 7 Imperial College, London, UK

**Keywords:** Patient and public involvement, Patient and public engagement, Cancer, Prostate cancer, Study design

## Abstract

**Background:**

ReIMAGINE aims to improve the current prostate specific antigen (PSA)/biopsy risk stratification for prostate cancer (PCa) and develop a new image-based method (with biomarkers) for diagnosing high/low risk PCa in men. ReIMAGINE’s varied patient and public involvement (PPI) and engagement (PE) strategy maximises the impact of its scientific output by informing and shaping the different stages of research.

**Aims:**

Through including the voice of patients and the public, the ReIMAGINE Consortium aims to translate these different perspectives into the design and implementation process. This will improve the overall quality of the research by:

- reflecting the needs and priorities of patients and the public, ensuring methods and procedures are feasible and appropriate
- ensuring information is relevant and accessible to those being recruited to the study
- identifying dissemination channels relevant to patients/the public and developing outputs that are accessible to a lay audience

With support from our patient/user groups, the ReIMAGINE Consortium aims to improve our ability to derive prognostic information and allocate men to the most appropriate and effective therapies, using a novel image-based risk stratification with investigation of non-imaging biomarkers.

**Findings:**

We have been working with patients and the public from initiation of the project to ensure that the research is relevant to men and their families. Our PPI Sub-Committee, led by a PCa patient, has been involved in our dissemination strategy, outreach activities, and study design recommendations. For example, the sub-committee have developed a variety of informative videos relevant and accessible to those being recruited, and organised multiple online research engagement events that are accessible to a lay audience. As quoted by one of the study participants, “the more we present the benefits and opportunities to patients and the public, the more research commitment we obtain, and the sooner critical clinical questions such as PCa diagnostics will be addressed”.

## Plain English Summary

One in eight men will be diagnosed with prostate cancer (PCa). Most will not die of it, but our ability to identify those men whose cancer poses the greatest threat to life has, thus far, been poor. Some men are diagnosed with small cancers which will never cause them a problem, some will have treatment which is unnecessary, others will have their cancers missed, and others will be misclassified as either having low risk cancer and will therefore miss out on the appropriate treatment, or told their cancer is high risk and have unnecessary treatment. Nowhere else in modern medicine are these errors of over-diagnosis, over-treatment, missed-diagnoses, and poor risk-stratification more common.

The ReIMAGINE Consortium has been developed to undertake discoveries that will correct these four key errors in the PCa diagnostic pathway. We will investigate how to best identify which men have, or will develop, aggressive prostate cancer using imaging combined with advanced biomarker analyses of blood and urine (i.e., OMICs technologies such as whole genome sequencing, targeted sequencing (e.g.: = , methylation). We will achieve this by building on established partnerships between patients, advocacy organisations, clinicians, imaging experts, molecular biologists, methodologists, and a broad range of industrial partners.

The Patient and Public Involvement (PPI) sub-committee is an integral part of the study workflow, contributing to study design and recruitment, results analysis, and dissemination. The committee, led by a funded PPI co-ordinator and a patient chair, have given invaluable insight into the study modifications due to COVID-19 restrictions.

## Background

ReIMAGINE, a consortium funded by the Medical Research Council (MRC) and Cancer Research UK (CRUK), was established in 2018 to further refine the use of magnetic resonance imaging (MRI), in combination with diagnostic molecular markers, as a tool for precise baseline risk stratification of men being assessed for prostate cancer (PCa). This work is needed as, unfortunately, for men with apparently low risk PCa, it is not yet possible to distinguish those tumours that will remain indolent and those that will develop into high risk or advanced disease [1].

ReIMAGINE aims to recruit 1,000 men referred to secondary care with a suspicion of PCa or men who are undergoing further tests for PCa staging into a prospective cohort study, with detailed assessment of baseline characteristics (i.e., clinic-demographics as well as (liquid) biopsies and imaging) and life-long follow-up (study A). In addition, Re-IMAGINE will run a pilot screening study aiming to evaluate MRI as a screening tool in 300 men (study B).

We have been working with patients and their families from initiation of the project and this has recently also led to recruitment completion for study B despite COVID-19 limitations (see below). Here, we describe how active patient and public involvement (working in partnership with patients and members of the public to plan, manage, design, and carry out research) and engagement (how information and knowledge about research is provided and disseminated) with patients and the public from inception leads to impactful evidence-based clinical research outputs for the ReIMAGINE Consortium and the studies within the project. It is important to point out that “PPI takes various forms, from involvement of public contributors in priority setting, representation on committees, and as reviewers” [2]. The latter 2 forms have been invaluable to our ongoing research processes.

### ReIMAGINE patient and public involvement (PPI) structure and processes

From the outset of this project, the ReIMAGINE Consortium operated on the premise of ‘no decision about the patient, without the patient’. The grant application was developed with both patient and public involvement [3, 4], and a patient was part of the team who were interviewed at the final stage of the application. During the grant writing/planning phase, we used a three-stage approach (i.e., information session, discussion groups, and feedback presentation), whereby we interacted with patients, their families, as well as men from the general population to develop an active partnership between patients and researchers [5, 6]. This collaboration between researchers and patients has identified and developed Re-IMAGINE in such a way that the output will be relevant and beneficial to patients and their needs.

Once funded, we appointed a PPI Coordinator to work within the PPI&E workstream. We began with a series of discussion groups, whereby patients and their family members, as well as men without PCa (i.e. the general public) provided insight into participant preferences with respect to design and management, data collection and analysis, and dissemination of findings. We had several meetings and ensured that the ethnic diversity of London was represented. The meetings were scheduled alternatively during the daytime and in the evening, to allow a wide range of patients and members of the public to engage. Following these discussion groups, we also presented the project for feedback to the South East London Consumer Research Panel and at the Guy’s Cancer Survivors Day (June 2017) for further feedback.

### Recommendations from PPI

The discussions confirmed the research need for less invasive diagnostic strategies for prostate cancer and generated study design recommendations. For instance, the participants of the discussion groups recommended us to speak with general practitioners (GPs) to ensure we had an efficient recruitment strategy for study B. A discussion group with 10 London-based GPs was therefore held to identify the best method for identifying community-based men eligible for study B. The GPs were asked about uptake and participation rates, the best method for contacting eligible participants, the possibility of collecting semen in addition to blood (something we then chose not to do), how to best inform the GP practice about the participants’ MRI results (especially when there is a possible PCa diagnosis), IT solutions, and reimbursements. All feedback was accounted for when submitting approval for the screening study. Over 10 GP Surgeries based in North, South and East London received approval to participate in this study.

In the context of our PPI and engagement strategy, we discussed project reporting, patient representation in the steering committee, the need for a PPI Sub-Committee, and a dissemination strategy. For the latter, we involved ecancer, one of the leading oncology communication charities. We were also encouraged to reach out to Prostate Cancer UK and Movember, two of the UK’s leading PCa patient support charities, which helped us to identify the chair for our PPI Sub-Committee. Mr Tuck was a Movember ambassador at the time and also leads a PCa patient support group in Oxfordshire. Hence, our discussion groups also provided an opportunity to build relationships with patient communities and involve individual patients going forward, as recommended by Doria et al. [7].

### Establishing the PPI sub-committee

Recruitment for PPI can often be challenging, however with the support of colleagues, such as, Prostate Cancer Clinical Nurse Specialist and Clinical Research Team we were able to find patients for the sub-committee. Such colleagues are a useful resource as they work directly with patients and have established relationships and can therefore suggest which patients might be willing to be involved in research. In addition, we also had the support of charities such as PCUK and Movember. Forming alliances with different teams/bodies has assisted us with our committee numbers and representation, working with our brief which specifies people of diverse backgrounds, in particular, men from the black community, given they are at a significantly increased risk of prostate cancer.​​​

The PPI Sub-Committee, chaired by Mr Tuck, meets consistently every three months to ensure that the needs and priorities of participants are practical and suitable as well as to ensure information is relevant and accessible to those being recruited and the public. The committee consists of two white men and one black man with prostate cancer between the ages of 40–80 years old, with varying educational qualifications. In addition, one black woman (age 43) whose father died of prostate cancer, the ReIMAGINE PPI Coordinator, and a research academic sit on the committee. The diversity of the group has permitted a greater range of perspectives from different age groups and communities to help shape the research. The Chair also attends fortnightly project meetings with the wider team and feeds back to the sub-committee accordingly and vice versa.

### The PPI sub-committee roles

Following the establishment of a terms of reference, outlining the aims and objectives of the group, the committee have reviewed all patient facing documents (e.g. patient information sheet, consent form, invitation letter), developed the patient area on the ReIMAGINE website, advising on design, layout and content. They created the storyboard and scripts for the patient information videos on the website by working closely with the PPI Coordinator and ecancer and engaged in the production process of the six-monthly newsletter through feedback on both content and design. The Chair also assists with editing the final draft of the newsletter.

### Prostate cancer specialist PPI sub- group

Additionally, through the active involvement and engagement of our PPI Sub-Committee, we have launched a specialist PCa Research Group for Black, Asian and Minority Ethnic communities, following the Black Lives Matter Movement in 2020. This specialist group provides a platform for a greater range of perspectives and allows researchers to access and foster dialogue between this under-represented population of patients and members of the public who wish to be involved with clinical research, in addition to providing an opportunity for their voices to be amplified through sharing their lived experiences – stimulating discussion and promoting greater diversity in research. We actively sought participation from men of diverse backgrounds and promoted our PPI group on Twitter, which has resulted in researchers from other institutions approaching us for support with their patient engagement activities. The ReIMAGINE PPI Coordinator was also selected to provide input for CRUK’s Equality, Diversity and Inclusion Strategy.

### ReIMAGINE outreach and engagement

**Fig. 1:**
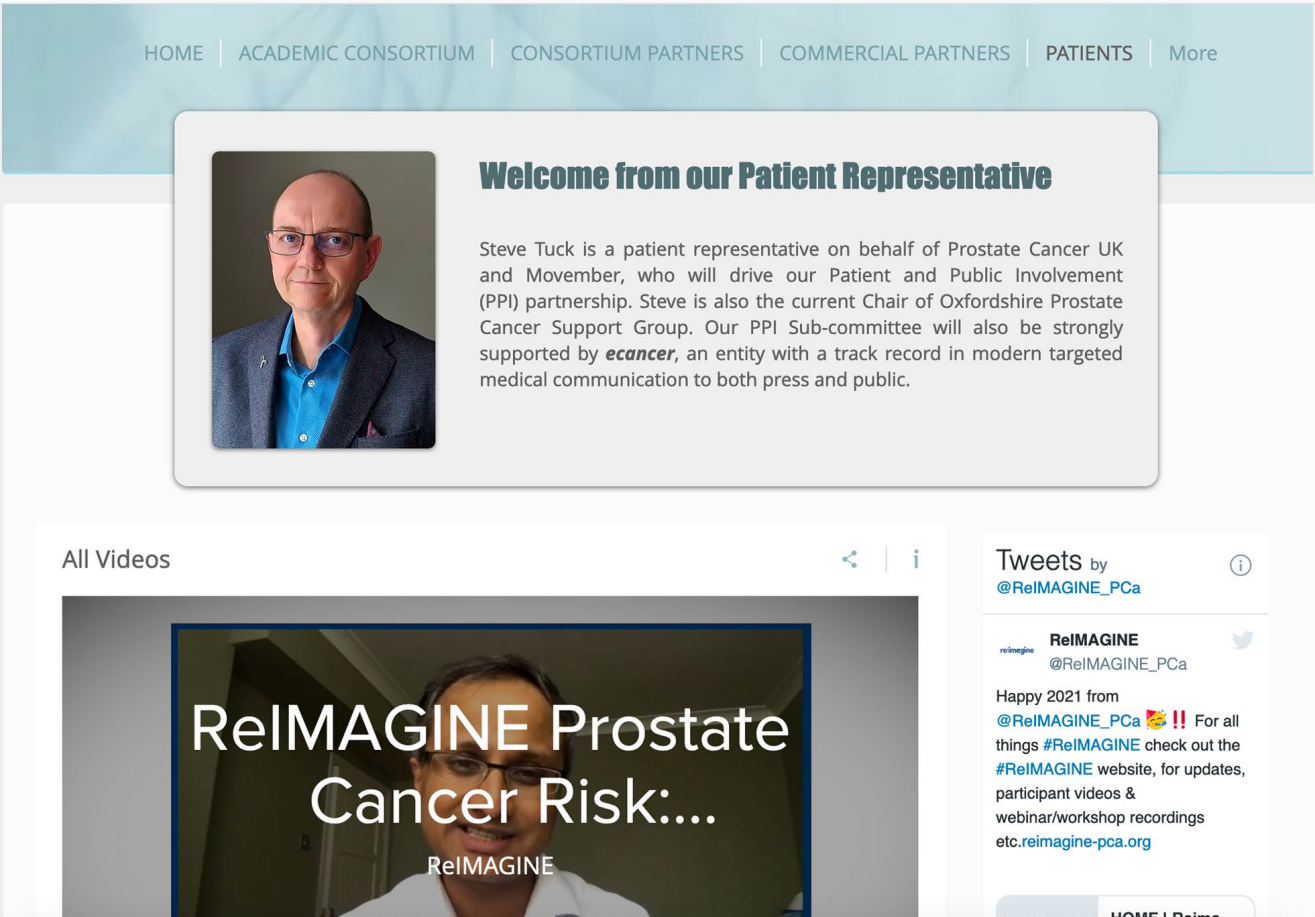
The patient area of the ReIMAGINE website has a dedicated section with videos (hosted on YouTube) to inform patients about the study processes and how participants will be recruited

The ReIMAGINE newsletter is written so that it is of interest to patients, the general public, healthcare professionals, and members of the ReIMAGINE Consortium. This patient-friendly update, as well as every other component of the PPI Sub-Committee’s dissemination strategy, is co-produced with ecancer who also supports the development of the videos, leaflets, flyers, banners, and invitations. The ReIMAGINE website has also been designed to be a source of information for researchers, healthcare professionals, and industry, as well as patients and the general public. The patient area has a dedicated section with videos (hosted on YouTube) to inform patients about the study processes and how participants will be recruited (Fig. 1). Members of the PPI Sub-Committee not only informed the content of these, but also participated as actors in the videos.

**Fig. 2:**
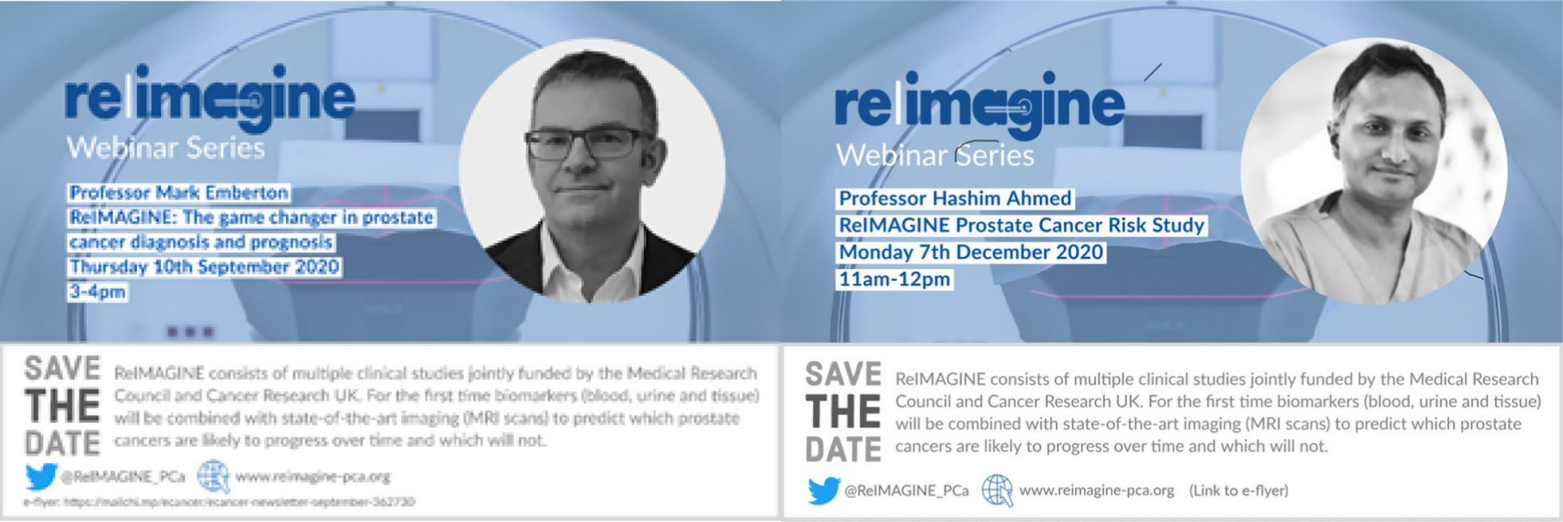
To further engage with the prostate cancer community outside of London, we have been organising free webinar series which were advertised widely with the help of CRUK

In addition, the PPI Sub-Committee engages in outreach activities throughout the UK to create awareness about PCa and the ReIMAGINE Consortium. Members advised on appropriate activities and what aspects of the study might be of interest to the public. Some members assisted with manning the stands on the respective days. For example, we have engaged in the International Clinical Trials Day at Guy’s Hospital (2019), an annual event where researchers have the opportunity to engage with patients, public and staff about their project; the Cancer Survivors’ Day at Guy’s Cancer Centre (2018 and 2019), a showcase of fashion, music and research via seminars and exhibits; and the Open Research Day of University College Hospital London (2018), a celebration of research; as well as participated in outreach activities of the Jamaica UK Diaspora in Birmingham. We also advertised a ReIMAGINE banner on the conference app of the 2019 Conference of the National Cancer Research Institute. To further engage with the prostate cancer community outside of London, we worked in partnership with charities and patient groups nationwide to promote our free webinar series (Fig. 2), which was moderated by our PPI Sub-committee Chair. In addition, each speaker’s presentation was reviewed by the Chair in order to ensure that the content was relevant and accessible to a lay audience. The PPI Sub-committee also produced a research article in one of the newsletters from Tackle – an umbrella organisation for > 90 PCa support groups in the UK. The PPI sub-committee members have played a crucial role in identifying different dissemination channels that are relevant to patients/the public and developing outputs that are accessible to a lay audience.

Twitter (@reimagine_pca) has been an invaluable platform for our research engagement as it allows us to participate in many other relevant PPI activities online: e.g. Urology Awareness month, World Cancer Research Day, Urology week, London Global Cancer Week, Prostate Cancer Awareness month, Black History Month, and International Men’s Day. The latter was also the day on which we had our first online research engagement event (19th November 2020) to inform the wider public about ReIMAGINE. This event was an evening of updates, interactive sessions and workshops with ReIMAGINE team members Prof Mark Emberton, Prof Hashim Ahmed, Prof Caroline Moore, and Prof Gerhardt Attard. The event also included an interview conducted by the PPI Sub-Committee chair with a participant. The PPI Sub-Committee was heavily involved in the organisation of this event, which was well received by the attendees. One prostate cancer patient representative who attended the event emailed to say that; “The webinar was ‘gold standard’ patient and public engagement. The particularly positive elements were: The session felt as if it had been co-produced with the PPI lead.

- Always positive to share results with patients and the public
- The agenda was well thought through
- The content was generally understandable to a lay audience and presentations were clear
- The men who’d participated in the study were thanked

“Your time commitment to this, shows your commitment to sharing research with lay audiences. Thank you. For me the more we present the benefits and opportunities research brings to patients and the public, the more we get commitment for them to be involved in future trials, the sooner we get answers to the critical research.”

### An example of our PPI impact: overcoming the challenges of COVID

Importantly, we would also like to report how our PPI strategy has helped ReIMAGINE overcome some of the research limitations due to COVID-19. In March 2020, NHS trusts and research sponsors implemented a variety of policies based on the interpretation of nationwide government sanctioned guidelines. Introduction of these policies resulted in the closure of all non-COVID related research from midway through that month. After a period of time NHS trusts and study sponsors developed processes of special exemption to reopen active recruitment. The Trial management groups worked closely with the PPI Sub-committee to ensure the modifications being implemented reflected the needs and priorities of patients and the public. Consequently, we developed a roadmap for reopening of the two studies. The committee members raised ideas that were not considered by the consortium, for instance, inclusion of COVID-19 specific and hygiene related information in the PIS/Invitation letter such as;

- how the MRI scanner is decontaminated
- no physical contact; no examination, no doctor present
- healthcare staff will have adequate PPE
- special provisions for BAME men, and men with comorbidities

In addition, we developed COVID-19 Safeguarding videos together with the PPI Sub-Committee and ecancer for both study A and study B, in which men clearly explained how we mitigated potential COVID-19 risks for those participating in ReIMAGINE. The PPI subcommittee also suggested study modifications to reassure men of the safety of participation, e.g. paid taxi travel in study B. This advice was not only useful in terms of its acceptability to the potential participants, but provided reassurance to the study sponsors, NHS trusts and National Research Ethics committees that study modifications were guided by patient opinion.

**Fig. 3:**
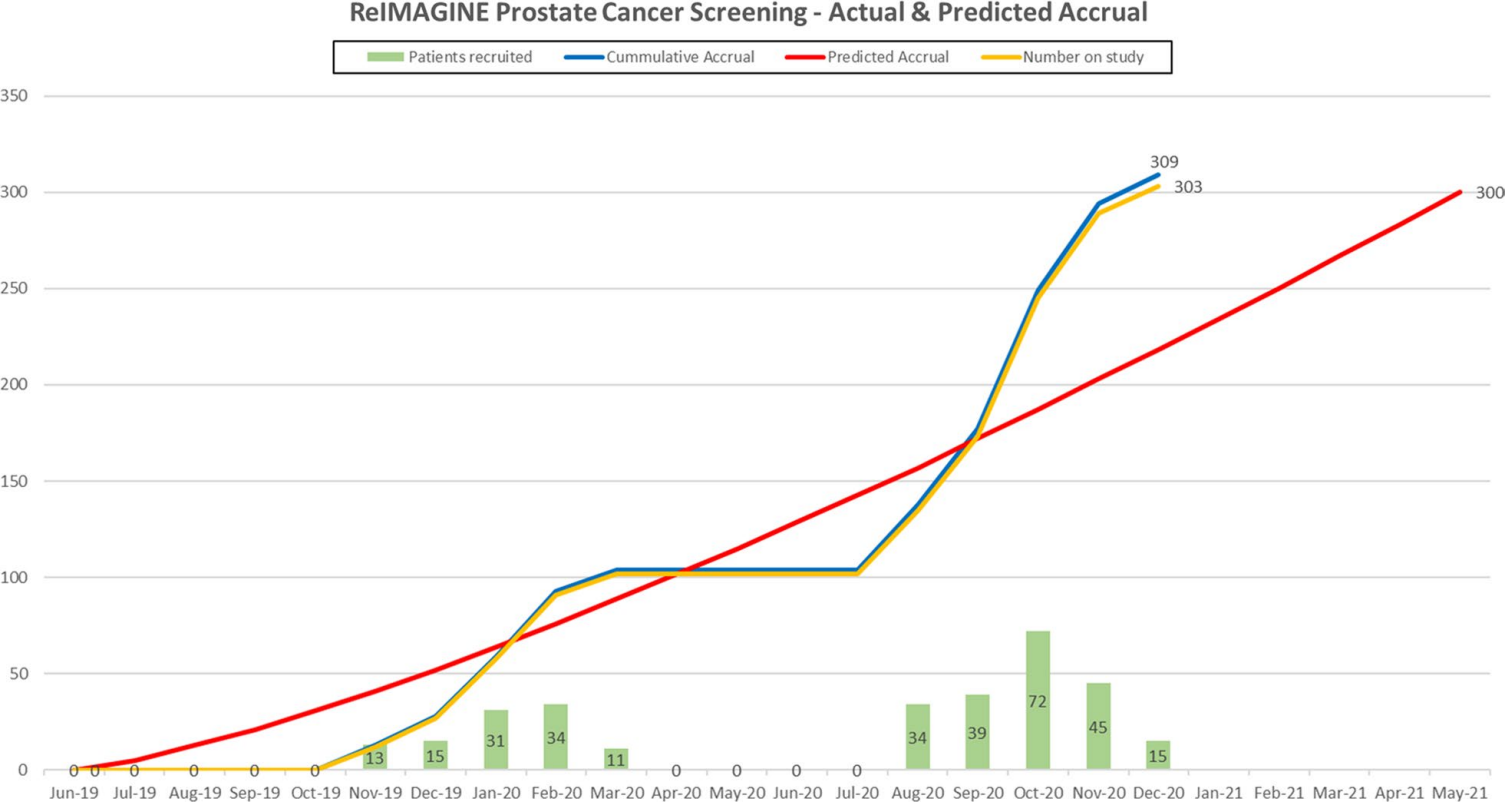
Recruitment rates of ReIMAGINE Study B before and during the COVID-19 pandemic

Recruitment for Study A is currently limited due to the ongoing COVID-surge in the UK, but Study B managed to recover impressive recruitment rates following the first COVID wave and completed recruitment in December 2020 (Fig. 3). Input from the PPI sub-committee has been invaluable, ensuring that appropriate modifications are in place to maximise patient, researcher and NHS safety. Thus increasing the number of potential patients and the uptake rate.

## Conclusions

The PPI strategy of ReIMAGINE ensures direct patient impact. The consortium has incorporated structures and funding for inclusion and engagement of the patient and public voice in the study design, monitoring and ongoing processes, resulting in a more effective and improved research process. The development of robust PPIE structures and processes that are embedded in the whole research process ensures that encountered challenges can be overcome demonstrated by the overcoming of recruitment challenges during the COVID-19 pandemic. The appointment of a funded PPI co-ordinator and a patient chair of the PPI sub-committee has led to further work outside the study remit, particularly in the establishment of a specialist PPI committee for PCa for people of diverse ethnic backgrounds.

## Data Availability

Not applicable.

## Abbreviations

BAME: Black, Asian and Minority Ethnic
GP: General practitioner
MRI: Magnetic resonance imaging
PCa: Prostate cancer
PIS: Patient information sheet
PPI: Patient and public involvement
